# Supplementary material for: Verifiable Agentic Science on Public Infrastructure
Source: bioRxiv. 2026 Oct 28:2026.10.26.761864. Preprint. [Version 1] doi: 10.64898/2026.10.26.761864 (PMC13733885; doi:10.64898/2026.10.26.761864)
Supplement: Supplementary file 1 [file NIHPP2026.10.26.761864V1-supplement-1.pdf]

bioRxiv preprint doi: https://doi.org/10.64898/2026.10.26.761864; this version posted October 28, 2026. The copyright holder for this preprint (which was not certified by peer review) is the author/funder, who has granted bioRxiv a license to display the preprint in perpetuity. It is made available under a CC-BY 4.0 International license.

Supplementary Information

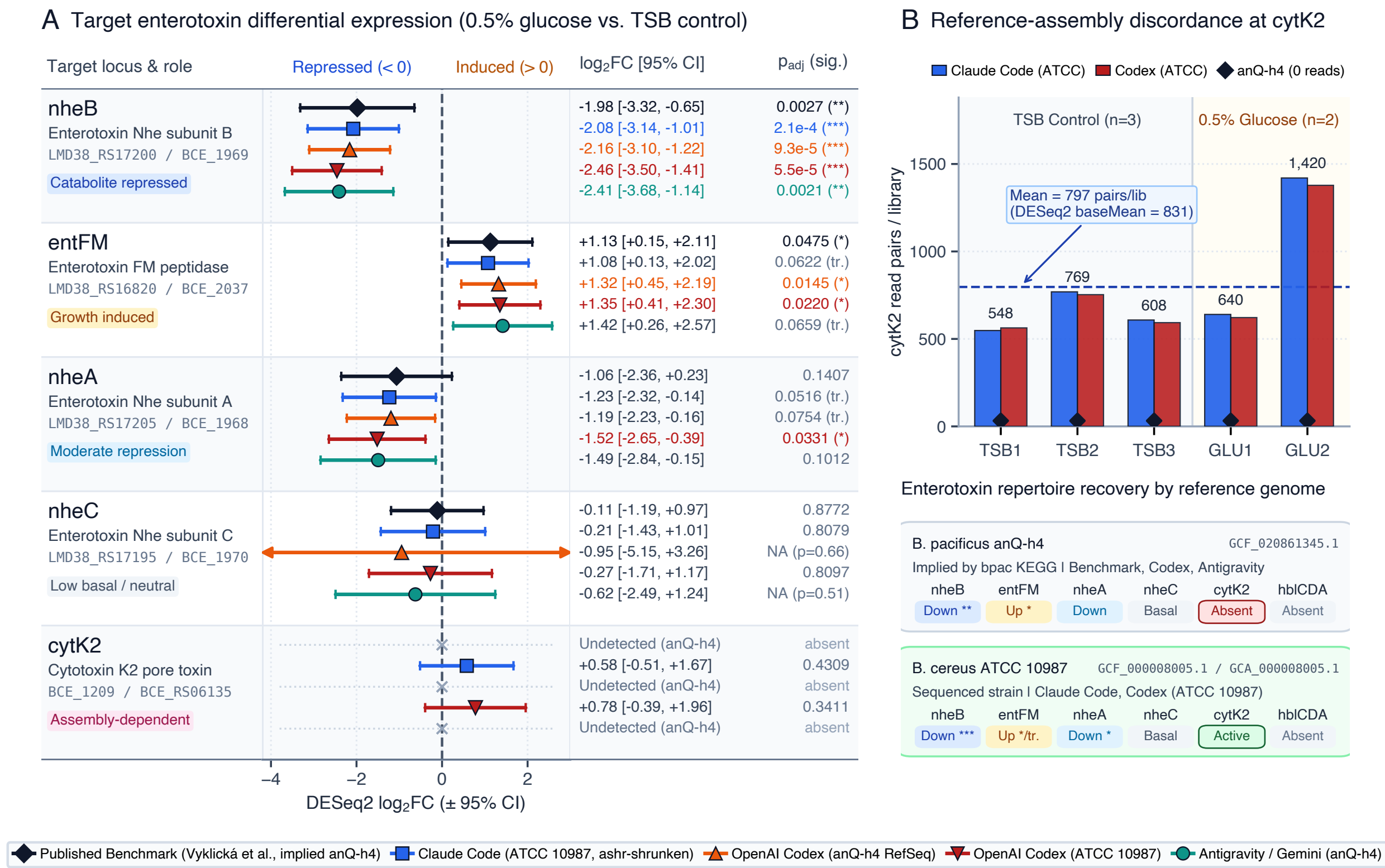

Figure S1: Local agent runs recover headline enterotoxin signals but diverge on reference genomes and leave unversioned glue code. A, Differential expression estimates ($\log_2$ FC with 95% confidence intervals) and adjusted p-values for target enterotoxin genes (*nheB*, *entFM*, *nheA*, *nheC*, and *cytK2*) compared against the published study (black diamonds, Vyklická et al. [15]). OpenAI Codex (orange and rust across two reference assemblies) and Google Gemini via Antigravity (teal) report unshrunken DESeq2 Wald estimates, whereas Claude Code (blue, `nf-core/differentialabundance`) reports `ashr`-shrunken estimates (for *nheC* on *B. pacificus*, where independent filtering sets p_{adj} to `NA`, the unadjusted p-value is shown). All three agents confirmed strong repression of *nheB* and induction of *entFM*. **B**, Reference-assembly mismatch at the cytotoxin K (*cytK2*) locus. Aligning against the sequenced isolate *B. cereus* ATCC 10987 in Claude Code and Codex recovered active baseline *cytK2* transcription across all libraries (548–1,420 read pairs per library, mean 797, DESeq2 baseMean = 831, $p_{\text{adj}} = 0.43$). Aligning against *B. pacificus* anQ-h4 missed *cytK2* entirely because that assembly lacks the locus.

bioRxiv preprint doi: https://doi.org/10.64898/2026.10.26.761864; this version posted October 28, 2026. The copyright holder for this preprint (which was not certified by peer review) is the author/funder, who has granted bioRxiv a license to display the preprint in perpetuity. It is made available under a CC-BY 4.0 International license.

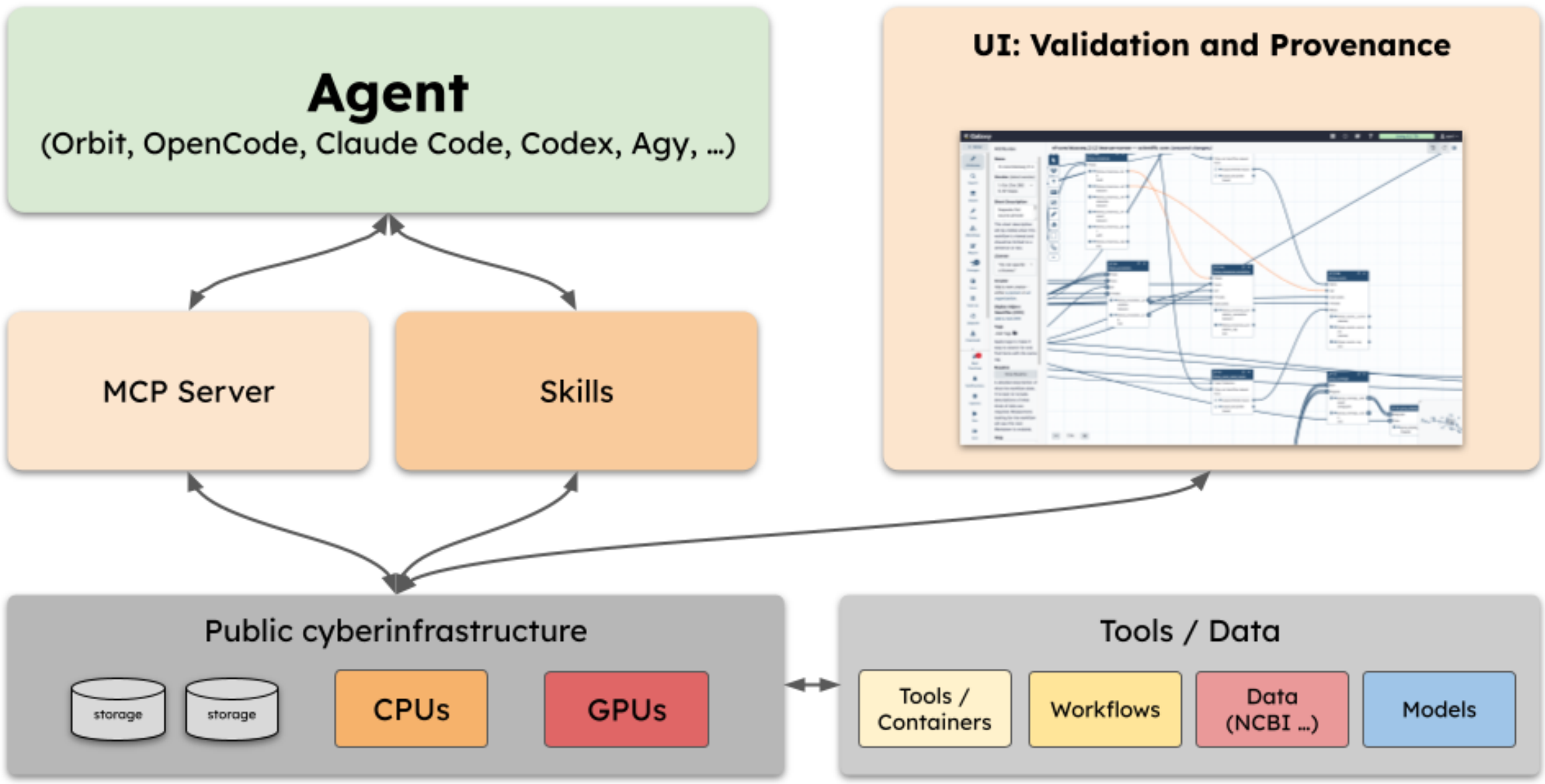

Figure S2: Architecture for verifiable agentic science on public infrastructure. Agent (top left): Researchers use Orbit or connect any generic coding agent (such as OpenCode, Claude Code, OpenAI Codex, or Antigravity CLI). **MCP Server and Skills** (middle left): Curated domain skills supply validated bioinformatics protocols, while the Galaxy MCP server translates agent actions into typed API calls in place of unconstrained shell commands. **UI: Validation and Provenance** (top right): Galaxy's visual workflow editor and history interface provide pre-execution schema validation and an inspectable provenance record for human steering and verification. **Public cyberinfrastructure and Tools/Data** (bottom): Galaxy schedules containerized tools, peer-reviewed workflows, public datasets (NCBI, ENA, BRC-Analytics), and large models across CPU, GPU, and storage allocations on national supercomputing infrastructure.

bioRxiv preprint doi: https://doi.org/10.64898/2026.10.26.761864; this version posted October 28, 2026. The copyright holder for this preprint (which was not certified by peer review) is the author/funder, who has granted bioRxiv a license to display the preprint in perpetuity. It is made available under a CC-BY 4.0 International license.

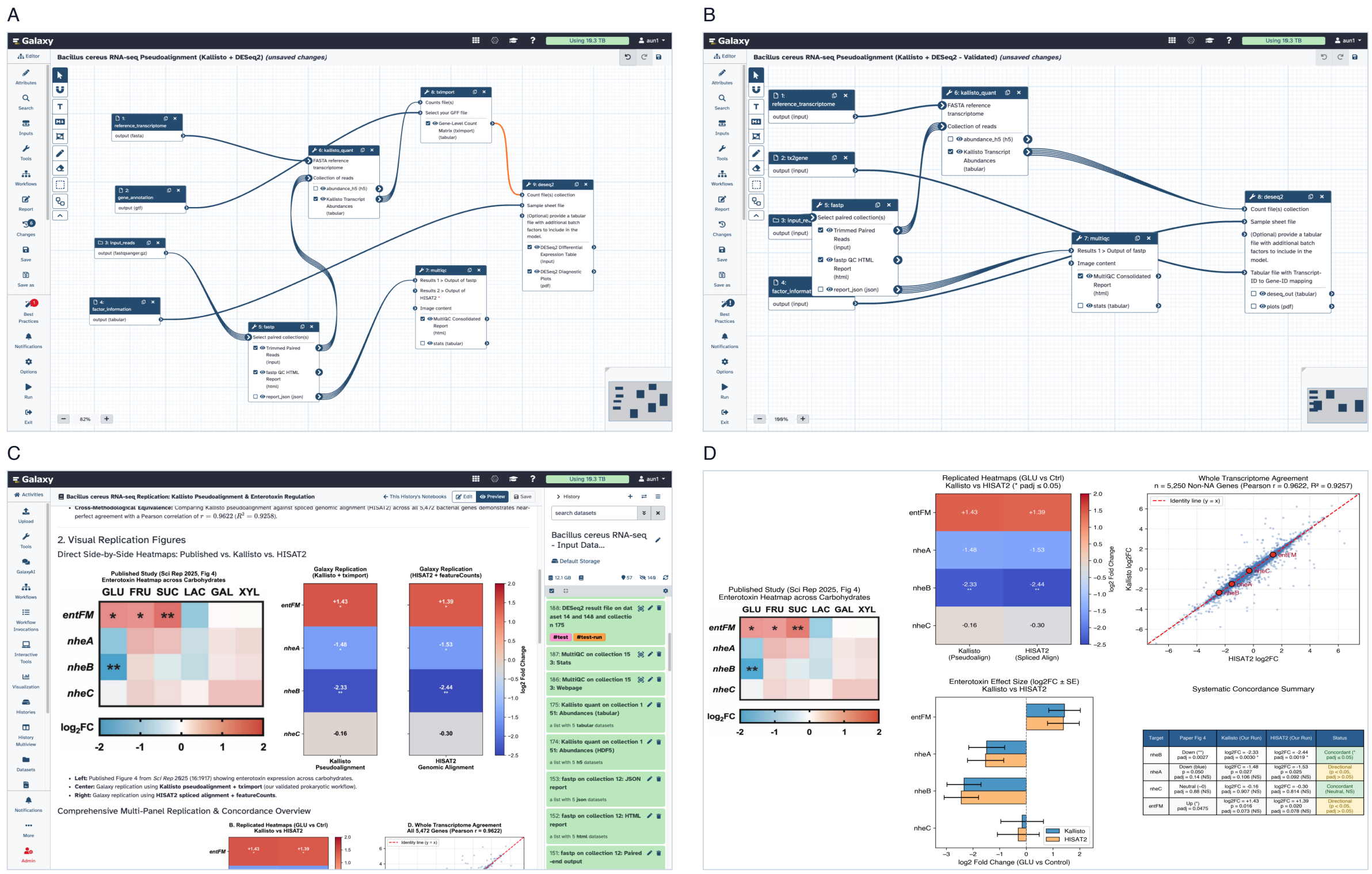

Figure S3: Workflow and execution provenance on free public infrastructure catches pipeline errors and reproduces bacterial RNA-seq profiles. A, Pre-execution workflow validation on UseGalaxy.org. When an agent connected via the Galaxy MCP server inserts an incompatible standalone `tximport` step between a Kallisto output collection and DESeq2, the workflow editor flags the type mismatch in orange. **B**, Validated workflow after repair. Passing the transcript-to-gene table to DESeq2's built-in `tximport` mode resolves all workflow connections in green. **C**, Live provenance and interactive Galaxy History Notebook. Jobs execute on free public ACCESS-CI supercomputing allocations while completed datasets populate the right-hand history panel and the center notebook links narrative text to verified history dataset IDs. **D**, Biological replication of Vyklická et al. [15] across the *B. cereus* ATCC 10987 coding transcriptome. Supplementation with 0.5% glucose represses enterotoxin subunits *nheB* (5.05-fold down, $\log_2 \text{FC} = -2.335$, $p_{\text{adj}} = 2.99 \times 10^{-3}$) and *nheA* (2.80-fold down, $\log_2 \text{FC} = -1.484$, raw $p = 0.0274$, $p_{\text{adj}} = 0.106$), leaves component *nheC* unchanged ($\log_2 \text{FC} = -0.158$, $p_{\text{adj}} = 0.907$), and induces *entFM* (2.70-fold up, $\log_2 \text{FC} = +1.432$, raw $p = 0.0158$, $p_{\text{adj}} = 0.0730$). Across all 5,250 expressed coding loci (of 5,472 total), Kallisto pseudoalignment and HISAT2 alignment agree closely (Pearson $r = 0.9622$, $R^2 = 0.9257$).

bioRxiv preprint doi: https://doi.org/10.64898/2026.10.26.761864; this version posted October 28, 2026. The copyright holder for this preprint (which was not certified by peer review) is the author/funder, who has granted bioRxiv a license to display the preprint in perpetuity. It is made available under a CC-BY 4.0 International license.

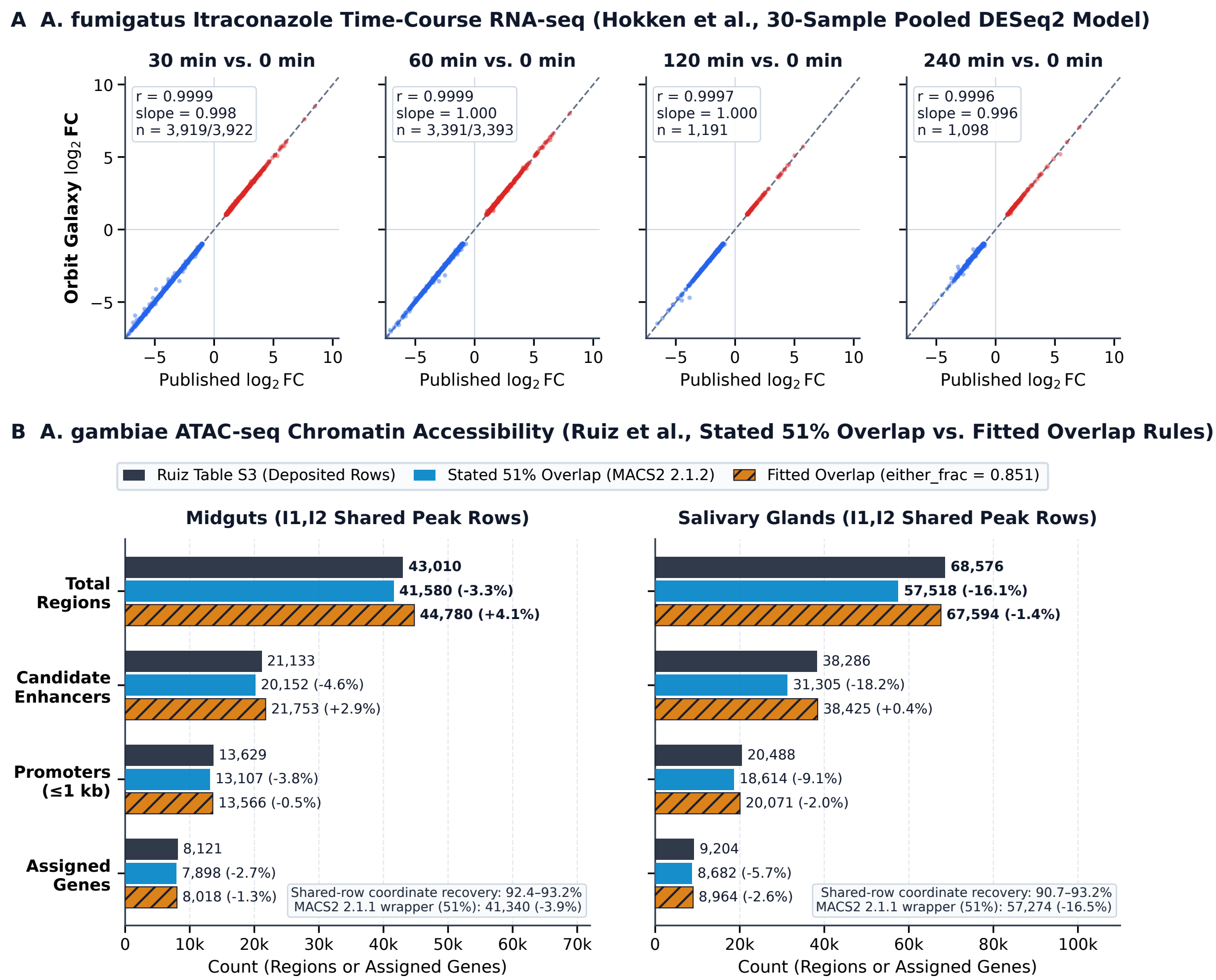

Figure S4: Reproducing pathogen and vector functional genomics studies on UseGalaxy.org. A, Transcriptomic response of the pathogenic mold *Aspergillus fumigatus* to the antifungal drug itraconazole across 30 libraries (Hokken et al. [26]). A pooled 30-sample DESeq2 model compiled via Workflow Foundry reproduces published $\log_2$ fold changes across all four post-exposure timepoints ($r = 0.9996$–0.9999, slopes 0.996–1.000). Published differentially expressed gene counts match within 0.3–1.3% ($n = 3,919$ of $3,922$ at 30 min, $3,391$ of $3,393$ at 60 min, $1,191$ at 120 min, and $1,098$ at 240 min). **B**, Chromatin accessibility in *Anopheles gambiae* malaria mosquito midguts and salivary glands during *Plasmodium falciparum* infection (Ruiz et al. [27]). Independent MACS2 peak calling recovers 90.7–93.2% of the 111,586 replicate-shared genomic regions in the authors' deposited table across all four libraries (81.8–92.2% across all 193,005 deposited peaks). Provenance-tracked overlap rules show how unmerged per-replicate rows and overlap filtering account for the headline region, promoter, and enhancer counts derived from Ruiz Table S3 within 0.4–2.9% (0.4–4.6% across tracks).

bioRxiv preprint doi: https://doi.org/10.64898/2026.10.26.761864; this version posted October 28, 2026. The copyright holder for this preprint (which was not certified by peer review) is the author/funder, who has granted bioRxiv a license to display the preprint in perpetuity. It is made available under a CC-BY 4.0 International license.

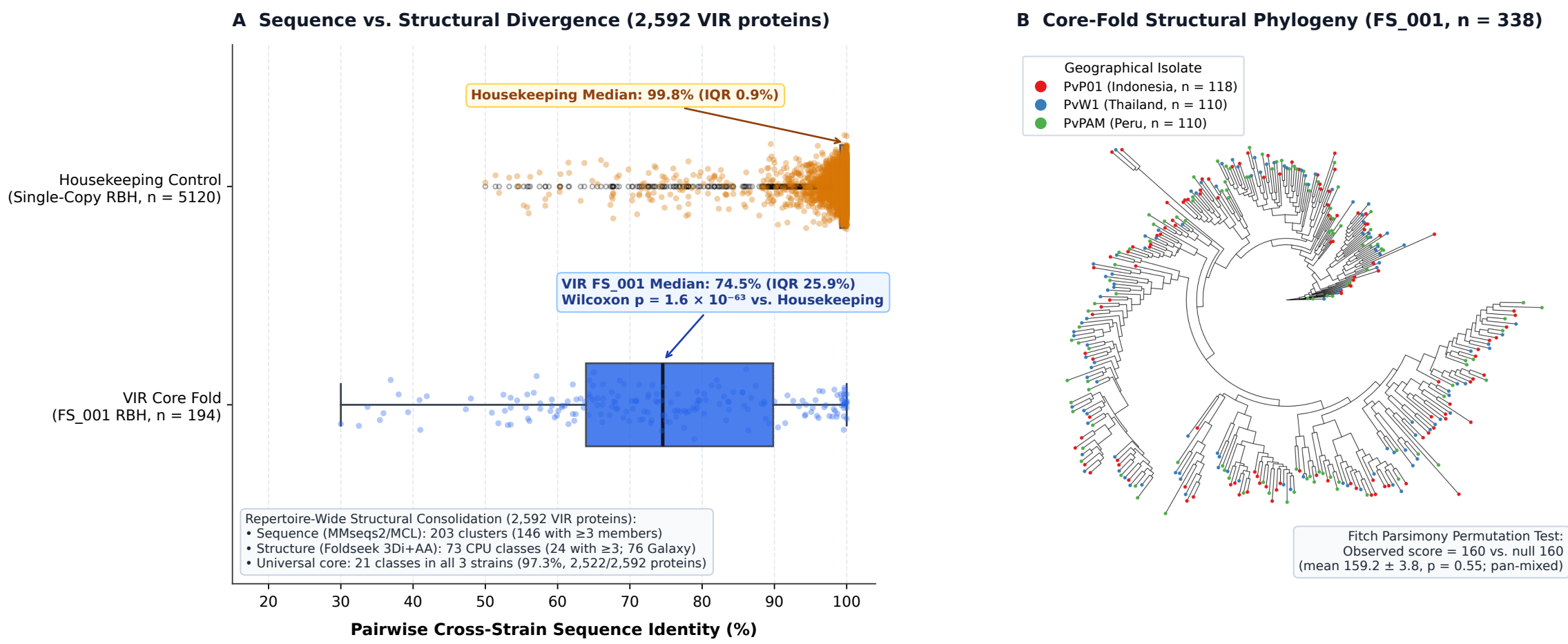

Figure S5: Structure-aware family reconstruction and lineage sharing of *Plasmodium vivax* VIR surface antigens across continental genomes. **A**, Sequence twilight zone and structural unification of over-split subfamilies. Horizontal box-and-strip plots display pairwise amino acid sequence identity between PvW1 (Thailand) and PvPAM (Peru) for conserved single-copy housekeeping genes (median 99.8%, IQR 0.9%, amber, $n = 5,120$ pairs) versus VIR pairs within dominant superfamily FS_001 (median 74.5%, IQR 25.9%, Wilcoxon $p = 1.6 \times 10^{-63}$, blue, $n = 194$ pairs). Sequence-only Markov clustering at 30% minimum sequence identity splits the 2,592 VIR proteins across Indonesia (PvP01), Thailand (PvW1), and Peru (PvPAM) into 203 sequence clusters (146 subfamilies with ≥ 3 members). Structure-aware clustering using ProstT5 and Foldseek 3Di representations collapses this repertoire into 73–76 structural classes (24–30 with ≥ 3 members) across local and UseGalaxy.org execution environments, where the dominant superfamily FS_001 unifies 89 sequence subfamilies on the local reference branch (see Methods). **B**, Circular maximum-likelihood structural phylogeny inferred by IQ-TREE 3 from FoldMason alignments of 338 FS_001 representatives on the local reference branch across Indonesia (PvP01, red, 34.9%), Thailand (PvW1, blue, 32.5%), and Peru (PvPAM, green, 32.5%) using partitioned amino acid and 3Di structural models. Continental lineages are interleaved across the tree, and a tip-label parsimony permutation test shows no detectable geographic clustering (observed score = 160 vs. 1,000 random shuffles with null median 160, $p = 0.55$).

bioRxiv preprint doi: https://doi.org/10.64898/2026.10.26.761864; this version posted October 28, 2026. The copyright holder for this preprint (which was not certified by peer review) is the author/funder, who has granted bioRxiv a license to display the preprint in perpetuity. It is made available under a CC-BY 4.0 International license.

Metric	Google Gemini 3.8 Flash (Antigravity)	Anthropic Claude Opus 5.5 (Claude Code)	OpenAI Codex / GPT-6 Astra (Codex CLI)
**Agent Runtime**	Antigravity CLI (`agy`) driving ad-hoc Bash and R scripts	Claude Code (`cc`) driving Nextflow workflows via Docker	Codex CLI (`codex exec`) driving modular Bash runner
**Software Stack**	Bowtie2 v2.5.5, featureCounts v2.1.1, DESeq2 v1.50.2, fastp v1.4.0	nf-core/rnaseq 3.14.0 (STAR, Salmon), nf-core/differentialabundance 1.4.0	HISAT2 v2.2.1, featureCounts, DESeq2, fastp v0.24.0, FastQC
**Thread & CPU Strategy**	Greedy 16-thread allocation across download, alignment, and quantification	Automated Docker scheduling across 24 host CPU cores	Strict budget: `OMP/MKL=1`, explicit slices (10 align, 3 sort, 2 QC, 4 count)
**Peak Memory (RAM)**	2–4 GB (standard heap allocation)	16–30 GB (STAR 2-pass indexing and alignment in Docker)	<6 GB (explicit buffer limit: `samtools sort -m 512M`)
**Disk Footprint**	15–20 GB (immediate inline cleanup of raw SRA and FASTQ files)	>100 GB scratch disk (49 GB Nextflow cache and >40 GB QC sorting files)	<20 GB (sequential processing, pruned FASTQs tracked in ledger)
**Dependency Model**	Shared host Conda environment (`bacillus_rnaseq`)	Local Conda environment plus Docker OCI container layers	Project-isolated cache (`.cache/`) with explicit package URLs
**Custom Scripts Generated**	3 scripts (1 Bash, 1 Python, 1 R, 302 LOC)	9 scripts (7 Bash, 1 Python, 1 R, 190 LOC)	14 scripts (6 Bash, 7 Python, 1 R, 774 LOC)
**Reference Strategy**	Single reference (*B. pacificus* anQ-h4 RefSeq)	Single reference (*B. cereus* ATCC 10987 GenBank/RefSeq)	Dual-reference sensitivity (*B. pacificus* anQ-h4 and ATCC 10987)
**Execution Friction**	None (completed without intervention)	Host disk exhaustion (required manual container deletion and skipping QC)	None (preventative buffer limits and resumption caching)

Table S1: Resource allocation, software environments, and compute footprints across three autonomous agent execution runs. All three runs re-analyzed the primary glucose versus control contrast from the *Bacillus cereus* transcriptomic benchmark (BioProject PRJEB86181) on an identical host machine (Ubuntu Linux workstation with 24 CPU cores, 62 GB RAM, and NVMe SSD storage). While all three platforms recovered the core biological signal of enterotoxin regulation, their resource management paradigms differed sharply. Gemini utilized a lightweight, greedy Unix shell pipeline with immediate file cleanup. Claude executed a heavy containerized Nextflow workflow that consumed over 100 GB of scratch disk and caused disk exhaustion until secondary quality control tools were disabled. Codex applied strict computational budgeting. It capped memory buffers and thread allocations per stream while maintaining an explicit ledger of purged intermediate files. None of the three runs produced persistent provenance records that external researchers could inspect or re-execute without rebuilding the local execution environment.

bioRxiv preprint doi: https://doi.org/10.64898/2026.10.26.761864; this version posted October 28, 2026. The copyright holder for this preprint (which was not certified by peer review) is the author/funder, who has granted bioRxiv a license to display the preprint in perpetuity. It is made available under a CC-BY 4.0 International license.

Agent Runtime	Custom Scripts	Script Breakdown and Roles	Execution Paradigm and Portability
**Google Gemini 3.8 Flash** (Antigravity CLI, `agy`)	**3 scripts** (302 LOC)<br>1 Bash<br>1 Python<br>1 R	`download_fastqs.py` (45 LOC): parallel ENA FTP fetch.<br>`run_pipeline.sh` (93 LOC): Bowtie2, fastp, featureCounts.<br>`run_deseq2.R` (164 LOC): DESeq2 Wald testing, PCA, plots.	Monolithic shell execution with hard-coded paths. Performs immediate cleanup of raw files but lacks checkpoint resumption or portable provenance.
**Anthropic Claude Opus 5.5** (Claude Code, `cc`)	**9 scripts** (190 LOC)<br>7 Bash<br>1 Python<br>1 R	**Driver:** `run_all.sh` (12 LOC), `env.sh` (2 LOC).<br>**Ref and Data:** `00_reference.sh` (16 LOC), `01_download.sh` (22 LOC), `02_make_gtf.py` (28 LOC).<br>**Workflows:** `03_rnaseq.sh` (15 LOC), `04_featurecounts.sh` (40 LOC), `05_differentialabundance.sh` (7 LOC), `06_target_genes.R` (48 LOC).	Wraps heavy containerized Nextflow pipelines with custom glue scripts. Managed disk cache resumption but experienced host storage exhaustion.
**OpenAI Codex** (GPT-6 Astra, Codex CLI)	**14 scripts** (774 LOC)<br>6 Bash<br>7 Python<br>1 R	**Driver and Env:** `run_all.sh` (17 LOC), `env.sh` (16 LOC), `status.py` (18 LOC).<br>**Data and Ref:** `fetch_metadata.py` (46 LOC), `download_sra.py` (42 LOC), `download_reads.py` (56 LOC), `convert_reads.sh` (21 LOC), `prepare_reference.py` (60 LOC).<br>**Compute:** `process_sample.sh` (58 LOC), `process_all.sh` (8 LOC), `diagnose_mapping.sh` (15 LOC), `count_and_qc.py` (105 LOC), `differential_expression.R` (137 LOC), `make_report.py` (175 LOC).	Modular Unix pipeline with defensive thread and buffer throttling. Logs pruned intermediates in a custom ledger but cannot be shared without rebuilding the local environment.
**All Runtimes Combined**	**26 scripts** (1,266 LOC)	14 Bash, 9 Python, 3 R	Zero shared scripts, zero common workflow definitions, and zero portable provenance across all three platforms.

Table S2: Summary of custom scripts generated by autonomous agents during empirical reanalysis. Number of custom scripts, programming languages, code volumes, and functional scopes generated by each runtime to replicate the *Bacillus cereus* RNA-seq benchmark. Gemini implemented a minimal three-script pipeline driving standard command-line utilities. Claude built nine wrapper and glue scripts to drive containerized nf-core workflows and parse downstream abundances. Codex constructed a modular fourteen-script suite featuring automated metadata queries, dual-reference mapping, explicit buffer throttling, and HTML dashboard synthesis. None of the twenty-six scripts were portable or auditable beyond the specific workstation where they were generated.

bioRxiv preprint doi: https://doi.org/10.64898/2026.10.26.761864; this version posted October 28, 2026. The copyright holder for this preprint (which was not certified by peer review) is the author/funder, who has granted bioRxiv a license to display the preprint in perpetuity. It is made available under a CC-BY 4.0 International license.

Study / System	Identifier / Link	Description
**Vyklická et al. (2025)** *Bacillus cereus* RNA-seq (PRJEB86181)	bbd44e69cb8906b59549f5524d3906f3 bbd44e69cb8906b5d979c5818e07f6ad ea7670a8c3fa3562 93fcddc692e0986f	Complete 5-library prokaryotic RNA-seq replication history on *B. cereus* ATCC 10987 (raw CDS HID 66, deduplicated CDS HID 106, `tx2gene.tsv` HID 148, Kallisto collection HID 175, DESeq2 results HID 188, and JupyterLab comparison notebook HID 192), initial HISAT2/featureCounts comparison history (HIDs 1–64), validated four-step Kallisto–DESeq2 integrated `tximport` workflow, and published interactive Galaxy Page.
**Hokken et al. (2019)** *Aspergillus fumigatus* RNA-seq (PRJNA482512)	bbd44e69cb8906b5a5cd735af575e8f5 b3638784a5d2aa43 b4346d545fb2b437	Complete 30-sample time-course history (pairwise DESeq2 fits HIDs 953–959, pooled 30-sample `~timepoint` model HIDs 961 and 4423), 60-step Galaxy workflow compiled via Workflow Foundry, workflow invocation, and three custom UDTs (`hokken_go_enrichment`, `hokken_venn_comparison`, and `hokken_revigo_summary`).
**Ruiz et al. (2021)** *Anopheles gambiae* ATAC-seq & RNA-seq (GSE152924)	bbd44e69cb8906b5e87b8ac73fb252a5 bbd44e69cb8906b57c33c7b295ddf477 bbd44e69cb8906b51645d526a9bd48d6 32f57ede003070c3, 6c3ed747eba652a6	Primary ATAC-seq peak-calling, two-sided 51% overlap, and `either_frac` cross-fit history, raw ATAC QC history, companion RNA-seq quantification history, two published ATAC-seq workflows, and five custom UDTs (`ruiz_tn5_fragment_extractor`, `ruiz_macs2_212_callpeak`, `ruiz_reproducible_ths`, `ruiz_either_frac_shared_peaks`, and `ruiz_either_frac_crossfit`).
**Wang et al. (2024)** *Aedes aegypti* scRNA-seq (SRR26591148-49)	https://usegalaxy.org/u/scottcain/p/wang-2024-aaegypti-midgut-scrna-seq-reanalysis https://usegalaxy.org/u/scottcain/h/wang-aaegypti-scrna-seq-veupathdb-annotation-rerun	Published Galaxy Page report and companion history containing STARsolo matrices, DropletUtils cell calls, custom `MT_`-prefixed GTF, Scanpy AnnData objects, and sample-blocked edgeR pseudobulk tables.

Table S3: Computational artifacts from the bacterial, fungal, and vector genomics reanalysis studies. Galaxy histories, workflows, User-Defined Tool definitions, and interactive Galaxy Pages archived on UseGalaxy.org.

bioRxiv preprint doi: https://doi.org/10.64898/2026.10.26.761864; this version posted October 28, 2026. The copyright holder for this preprint (which was not certified by peer review) is the author/funder, who has granted bioRxiv a license to display the preprint in perpetuity. It is made available under a CC-BY 4.0 International license.

Artifact Type	Identifier / Link	Description
Primary History	bbd44e69cb8906b596a6dfeaa66d7b35	Reproduction of published *Acta2* 1,000-cell tutorial subset
Full Dataset History	bbd44e69cb8906b5e19946d9d8770575	Full 33-gene spatial evaluation on Dataset 15 (public)
Single-Gene Workflow	fde4525ee8a0c99f	Executable workflow for target gene prediction
Full Evaluation Workflow	63012fffa29eb7bb	Published 10-fold cross-validation workflow across 33 genes
Workflow Invocation	7e806872876f7fb0	Terminal execution trace for *Acta2* prediction
User-Defined Tools	`tissue_spatial_prediction` v1.2.0, `tissue_full_evaluation` v1.0.0	Containerized TISSUE UDT specifications
Interactive Pages	14ebc62e2d1afb49, 787090c6bb87743a	Research notebook and evaluation report on UseGalaxy.org
Code Repository	https://github.com/nekrut/spatTrOrbit	Scripts, local references, and workflow templates

Table S4: Computational artifacts from the spatial transcriptomics reproduction study. Galaxy histories, workflows, UDT definitions, and execution traces archived on UseGalaxy.org and GitHub.

bioRxiv preprint doi: https://doi.org/10.64898/2026.10.26.761864; this version posted October 28, 2026. The copyright holder for this preprint (which was not certified by peer review) is the author/funder, who has granted bioRxiv a license to display the preprint in perpetuity. It is made available under a CC-BY 4.0 International license.

Artifact Type	Identifier / Link	Description
Primary Galaxy History	bbd44e69cb8906b5c0a92f1d9b046fdd	Multi-step phylogenomics execution and UDT routing trace on UseGalaxy.org
GPU & CPU Clustering UDTs	anton-gpu-xl-foldseek_prostt5_clustering, anton-32-foldseek_prostt5_clustering	Foldseek + ProstT5 3Di embedding routed to NVIDIA A100 GPU and 32-core CPU nodes
Cluster Extraction UDT	anton-1-foldseek_cluster_extract	Superfamily member extraction from Foldseek clustering tables
Structural Alignment UDT	anton-32-foldmason_align	32-core FoldMason lockstep AA + 3Di MSA in custom ProstT5 container
Pseudogene Rescue UDT	anton-1-gff_pseudogene_translator	Six-frame GFF3 pseudogene translation for PF05795 HMM screening
Orthology & Synteny UDTs	anton-1-step7_prepare_fastas, anton-8-mmseqs_easy_rbh_pair, anton-1-microsynteny_filter	Proteome partitioning, MMseqs2 reciprocal best hits, and flanking microsynteny scoring
Spatial & Phylo Prep UDTs	anton-1-spatial_analysis, anton-1-iqtree_structural_prep	Subtelomeric enrichment, label permutation tests, and partitioned IQ-TREE matrix preparation
Custom Container Image	quay.io/nekrut/foldmason-prostt5:4.dd3c235-cpu	Purpose-built ProstT5-enabled FoldMason container
Code Repository	https://github.com/nekrut/phist	Pipeline command ledger, 10 UDT specifications, and local reference outputs

Table S5: Computational artifacts from the *Plasmodium vivax* phylogenomics study. Galaxy histories, ten User-Defined Tool definitions, custom container images, and verification suites archived on UseGalaxy.org, Quay.io, and GitHub.

bioRxiv preprint doi: https://doi.org/10.64898/2026.10.26.761864; this version posted October 28, 2026. The copyright holder for this preprint (which was not certified by peer review) is the author/funder, who has granted bioRxiv a license to display the preprint in perpetuity. It is made available under a CC-BY 4.0 International license.

Artifact Type	Identifier / Link	Description
Preparation History	bbd44e69cb8906b5c7c1fd5515b95301	Input validation and collection preparation for 42 cohorts
Analysis History	bbd44e69cb8906b55342b6485eac77f7	End-to-end tree-free dating and diagnostics execution
Originals Archive	f9cad7b01a472135e65a92a49dc98cbe	Public binary archive of 42 author BEAST XML files
Batch Preparator UDT	`chronaeon_batch_preparator` v1.0.0	XML parser, FASTA extractor, and date validator
Cohort Analysis UDT	`chronaeon_cohort_analysis` v3.2.2	Tree-free dating, spline clock fitting, and LOOCV validation
Input Collections	e2f9a561d7c485b5 (XML), 25b287f1e09c05f2 (FASTA), 73f4b0a869dae7a4 (dates)	Synchronized 42-element BEAST XML, aligned FASTA, and tip-date collections
Output Collections	eacb6fa1da5e5cbe, c2c3f23ed16816c8	Dating JSON and diagnostic plot collections for 42 cohorts
Interactive Page	feee8a2c33d4f9e1	Published compendium evaluation report and concordance dashboard
Code, Prompts & Provenance	https://github.com/veg/chronaeon, `manuscript/prompts/`, `manuscript/figures/data/chronaeon/`	Verbatim Orbit harvesting/analysis prompts (`prompt.md`, `prompt-galaxy.md`, `prompt-galaxy-analysis.md`), benchmark TSVs, and comparator provenance table (`chronaeon_42cohorts_provenance.tsv`)

Table S6: Computational artifacts from the 42-pathogen molecular clock benchmark study. Galaxy histories, User-Defined Tool definitions, dataset collections, and published interactive reports archived on UseGalaxy.org and GitHub.

bioRxiv preprint doi: https://doi.org/10.64898/2026.10.26.761864; this version posted October 28, 2026. The copyright holder for this preprint (which was not certified by peer review) is the author/funder, who has granted bioRxiv a license to display the preprint in perpetuity. It is made available under a CC-BY 4.0 International license.

Comparator Provenance Class	Alignments	Cohort Identifiers (00–41)	Concordance & Median Error Summary
**Explicit Published 95% HPD / BCI** (Primary paper text or table reports root median and 95% HPD/BCI directly)	15 scored (+1 unscored in runner)	00 (Gire et al. 2014 81-seq root), 01, 03, 08, 09, 12, 14, 15, 22, 27, 28, 31, 32, 34, 37 (plus 39_chikv_reunion_dellicour2020: 2004.8 [2004.5, 2005.1] in published_comparators.tsv)	**10 / 15 (66.7%)** concordant under tree-free OLS/Spline (or **10 / 16 [62.5%]** including 39_chikv_reunion_dellicour2020). Includes 9 acute outbreaks with $\lvert\Delta t_{\mathrm{MRCA}}\rvert < 1.0$ yr (00, 01, 03, 08, 09, 15, 22, 28, 32) and Influenza H3N2 (12, interval overlap).
**Reconstructed Reference Interval** (Interval bracketed from prose origin statement, clock rate, or Skygrid cutoff)	24 scored (23 unique)	02, 04, 05, 06, 07, 10, 11, 13, 16, 17, 18, 19, 20, 21, 23, 24, 25, 26, 29, 30, 33, 35, 36, 41 (41_skygrid_rabies_gill2020, paired 14.5 kb coding partition of 00)	**14 / 24 (58.3%)** concordant under tree-free OLS/Spline (**13 / 23 [56.5%]** unique), rising to **15 / 24 (62.5%)** (**14 / 23 [60.9%]** unique) with HyphAeon Attention PGLS on Mpox Clade Ib (30).
**All Numeric Comparators Combined** (aggregate_benchmark_results.tsv)	39 scored (38 unique)	Cohorts 00–37 plus 41	**Tree-free OLS/Spline:** 24 / 39 (61.5%, or 57.1% of 42) raw, 23 / 38 (60.5%, or 56.1% of 41) unique (13 with $\lvert\Delta t_{\mathrm{MRCA}}\rvert < 1.0$ yr [12 unique] + 11 by interval overlap). Median $\lvert\Delta t_{\mathrm{MRCA}}\rvert = 7.19$ yr overall (8.32 yr unique) and 0.51 yr across 19 post-1980 cohorts (0.65 yr across 18 unique).<br>**With Mpox PGLS:** 25 / 39 (64.1%, or 59.5% of 42) raw, 24 / 38 (63.2%, or 58.5% of 41) unique. Median $\lvert\Delta t_{\mathrm{MRCA}}\rvert = 6.41$ yr overall (6.80 yr unique) and 0.40 yr post-1980 (0.46 yr unique).
**Unscored in Automated Runner** (NO_COMPARATOR in aggregate_benchmark_results.tsv)	3 unscored	38_mab_commins2023 (*M. abscessus*, prose ∼ 1960–1980 vs. 1975.87 [1966.21, 1982.46]), 39_chikv_reunion_dellicour2020 (CHIKV Reunion, published 2004.8 [2004.5, 2005.1] vs. 1954.99 [1948.09, 1960.62]), 40_hiv1_crf01ae_philippines2024 (HIV-1 CRF01_AE, prose ∼ 1995–2002 vs. 1975.84 [1969.29, 1980.93])	Left unscored in aggregate_benchmark_results.tsv. Against the auxiliary entries in published_comparators.tsv, 38_mab_commins2023 falls inside its prose clade range (∼ 1960–1980), whereas 39 and 40 infer pre-epidemic ancestral roots earlier than the focal outbreak introductions.

Table S7: Provenance classification and concordance breakdown of reference comparators across the 42 ChronAeon pathogen benchmark alignments. Per-cohort estimates, Fieller 95% confidence intervals, comparator sources, and single-pass versus multi-model values are archived in manuscript/figures/data/chronaeon/chronaeon_42cohorts_provenance.tsv.
